# The Current Status of Radiological Clinical Audit and Feedback on the ESR Guide to Clinical Audit in Radiology and the ESR Clinical Audit Tool (Esperanto) – an ESR Survey of European Radiology Departments

**DOI:** 10.1186/s13244-020-00843-0

**Published:** 2020-02-26

**Authors:** European Society of Radiology (ESR), David C. Howlett, Chinnoi Law, Adrian P. Brady, Lorenzo Bonomo, Andrea Rockall, Guy Frija

**Affiliations:** Vienna, Austria

**Keywords:** Clinical audit, Basic Safety Standards Directive (BSSD), Radiation protection, Internal audit, External direction, Radiology

## Abstract

Clinical audit “according to national procedures” is a legal requirement as defined within the recently implemented European Council Basic Safety Standards Directive (BSSD), 2013/59/Euratom. A survey was undertaken in 2019 to assess the current status of clinical audit in European radiology departments and for feedback on the recently published “ESR Guide to Clinical Audit in Radiology” and the “ESR Clinical Audit Tool (Esperanto)”. The survey was distributed within the European Society of Radiology (ESR) EuroSafe Imaging Star network and also to European national radiological societies which are institutional members of the ESR.

A total of 47/116 (41%) EuroSafe Imaging Star departments responded, and responses were received from 43 radiology departments from 16/48 national radiological societies.

Survey findings demonstrated a low awareness of recent key ESR audit-related publications (Esperanto), a lack of clinical audit infrastructure for BSSD compliance and by inference a poorly developed local/national communication and audit infrastructure in many cases.

Key stakeholders, including the ESR and the European national radiological societies, will need to continue to work with other bodies to further promote, integrate and to encourage resourcing of clinical audit at all levels, facilitating BSSD compliance and improving patient care.

## Key points

- Effective internal and externally directed departmental audit requires a functional and adequately resourced supporting infrastructure.
- There is relatively poor awareness of ESR audit related initiatives and an underdeveloped BSSD-related clinical audit infrastructure in many departments.
- Survey results suggest that existing mechanisms for external direction of local departmental audit by national societies or professional bodies will require significant resource allocation to maximise effectiveness.

## Patient Summary

Clinical audit means the continuous evaluation and improvement of processes and workflows in patient care, and should help caregivers to deliver their services according to standards. The purpose of this ESR survey was to evaluate the status of clinical audit systems in European radiology departments as well as to gather feedback on recent guidance publications in that field, and to assess the problems faced by radiological departments.

The updated BSSD (Basic Safety Standards Directive) has important implications for radiology departments in relation to radiation protection standards. The BSSD requires implementing clinical audits “in accordance with national procedures”. Compliance with the BSSD and use of clinical audit is a legal requirement, and also good clinical practice.

This survey among European radiology departments revealed that awareness of ESR audit-related initiatives was relatively poor and that there is a general lack of existing clinical audit infrastructures and processes.

In conclusion, the survey results suggest to national societies and professional bodies a significant investment in development, improvement and establishment of clinical audit infrastructures in local departmental audits. The ESR, its national radiological and other medical specialist societies will play a key role to continue to promote and integrate clinical audit into European radiological practice, but also to stress the importance of clinical audit at departmental and governmental level.

## Introduction

The European Basic Safety Standards Directive 2013/59/Euratom [1] was adopted by the Council of the European Union (CEU) in 2013, for transposition into the national legislation of EU Member States by February 2018. The updated BSSD has important implications for radiology departments across Europe in terms of reinforcing or establishing a broad range of required radiation protection standards. The BSSD also mandates carrying out clinical audit ‘in accordance with national procedures’. Radiology departmental compliance with the BSSD and demonstration of supporting processes of clinical audit is therefore not only good clinical practice, but also a legal requirement following 2018 BSSD implementation.

The European Commission has previously produced comprehensive guidance on clinical audit in medical practice [2]. Supporting and promoting good clinical audit in radiology is also a high priority for the European Society of Radiology (ESR), important ESR audit related initiatives include; the production of an updated clinical audit guide and toolkit (Esperanto) [3, 4]; the EuroSafe Imaging Call for Action [5]; an increase in higher profile dedicated audit-related educational sessions at the European Congress of Radiology (ECR 2018 - 2020).

To facilitate high quality and effective clinical audit requires the establishment of clinical audit systems and processes at both departmental (internal) and also at national (external) levels. The external direction of internal audit can be undertaken by suitable national professional bodies/societies and can enhance the quality of departmental level audit practice.

The ESR recently conducted and published surveys looking at a) uptake of BSSD requirements within the ESR EuroSafe Imaging Star network with a focus on supporting clinical audit [6] and b) the status of clinical audit and supporting infrastructure amongst European National Radiological Societies [7]. These surveys demonstrated a generalised lack of compliance with BSSD radiation protection and clinical audit requirements at departmental level and shortfalls in necessary clinical audit resourcing and infrastructure at national level.

This paper describes the results of a survey undertaken on behalf of the ESR examining the current status of radiological clinical audit in Europe and also requesting feedback on the 2019 ESR Guide to Clinical Audit in Radiology and the ESR Clinical Audit Tool (Esperanto) at departmental level. This survey differs in emphasis from the previously published surveys alluded to above [6, 7] – these surveys examined BSSD compliance at departmental level and clinical audit infrastructure at national level.

The aims of the survey:

- Evaluation of existing radiology departmental clinical audit systems and processes.
- To establish levels of awareness around the Esperanto publication and obtain end user feedback on the Esperanto audit templates/toolkit.
- Assess challenges at departmental level preventing clinical audit infrastructure implementation and development.
- To indirectly evaluate the functionality of communication/feedback mechanisms between National Radiological Societies and their national radiology departments.

## Materials and Methods

A survey was prepared using SurveyMonkey; the questions are included, with results, in Tables 1 and 2. The survey included a free text/comments section at the end. The survey questionnaire was distributed via the ESR Office using 2 separate mechanisms of distribution to 2 differing target audiences (survey questions broadly similar, see tables):

- The initial survey was distributed to all member societies within the well-established ESR National Radiological Societies network (Table 1). The survey was sent out on May 22^nd^, 2019 and closed (following a reminder) on July 12^th^, 2019. The National Radiological Societies were requested, in the email accompanying the survey, to distribute the enclosed survey to all radiology departments in their country, using their usual means of departmental communication, with data return directly from their radiology departments to the ESR Office.
- The second version of the same survey (minus the one question pertaining to EuroSafe Imaging Star network membership) was distributed to all departments within the ESR EuroSafe Imaging Star network (Table 2). The survey was sent out on July 3^rd^, 2019 and closed (following a reminder) on September 12^th^, 2019, data return directly to the ESR Office.

**Table 1:** Feedback Survey - Radiology Departments following contact via their National Radiological Society

Q1	Yes	No	No - application in process	I don't know							
Is your department a member of the ESR EuroSafe Imaging Stars network?	6 (13.95%)	28 (65.12%)	1 (2.33%)	8 (18.60%)							
Q2	Yes	No									
Are you familiar with the ESR publication - Esperanto 2019, the ESR Guide to Clinical Audit in Radiology and the ESR Clinical Audit Tool?	24 (55.81%)	19 (44.719%)									
Q3	Word of mouth /colleagues	ECR 2019	ESR email /twitter / publication	National radiology society	Other						
How did you hear about Esperanto 2019?	6 (23.08%)	5 (19.23%)	10 (38.46%)	3 (11.54%)	2 (7.69%)						
Q4	Clinical relevance	Readability	Accessibility								
Please grade the Esperanto 2019 document according to the following criteria (scale 1 = not useful, 10 = very useful)	25 (see table below*)	24 (see table below*)	24 (see table below*)								
Q5	Clinical utility	Usability	Range of topics								
Please grade the Esperanto 2019 templates according to the following criteria (scale 1 = not useful, 10 = very useful)	24 (see table below**)	24 (see table below**)	24 (see table below**)								
Q6	Yes	No	In development								
Does your radiology department engage in clinical audit - with a programme of clinical audit in place where findings, actions and re-audits are documented?	22 (51.16%)	9 (20.93%)	12 (27.91%)								
Q7	Yes	No									
Do you have a nominated department lead for clinical audit?	25 (58.14%)	18 (41.86%)									
Q8	Administrative support		Infrastructure /IT support		Clinical leadership		Clinical Engagement		Managerial support		
Which of the following local requirements for effective clinical audit are present in your department?	Yes	No	Yes	No	Yes	No	Yes	No	Yes	No	
	29 (69.05%)	13 (30.95%)	30 (71.43%)	12 (28.57%)	36 (85.71%)	6 (14.29%)	34 (80.95%)	8 (19.05%)	25 (59.52%)	17 (40.48%)	
Q9	Yes	No	In development								
Currently, does your department have a clinical audit structure in place allowing compliance with the EU-BSS?	11 (25.58%)	15 (34.88%)	17 (39.53%)								
Q10	Yes - exists, participate	No - exists, do not participate	No - does not exist								
Does your department participate in a national radiology audit programme?	27 (62.79%)	4 (9.30%)	12 (27.91%)								
Q11	National radiology society	Governmental body	Other national society	Other							
How is the national audit programme organised?	15 (34.88%)	14 (32.56%)	3 (6.98%)	11 (25.58%)							
Q12	Yes	Yes - but additional resources required	No								
In principle, would your department be prepared to participate in pan-European surveys/audits co-ordinated by the ESR/national radiology societies?	11 (25.58%)	20 (46.51%)	12 (27.91%)								
*	1	2	3	4	5	6	7	8	9	10	TOTAL
Clinical relevance	1 (4.00%)	0 (0.00%)	0 (0.00%)	0 (0.00%)	2 (8.00%)	1 (4.00%)	3 (12.00%)	10 (40.00%)	4 (16.00%)	4 (16.00%)	25
Readibility	1 (4.17%)	0 (0.00%)	0 (0.00%)	0 (0.00%)	0 (0.00%)	0 (0.00%)	5 (20.83%)	11 (45.83%)	6 (25.00%)	1 (4.17%)	24
Accessibility	1 (4.17%)	0 (0.00%)	0 (0.00%)	0 (0.00%)	1 (4.17%)	0 (0.00%)	3 (12.50%)	7 (29.17%)	6 (25.00%)	6 (25.00%)	24
**	1	2	3	4	5	6	7	8	9	10	TOTAL
Clinical utility	0 (0.00%)	1 (4.17%)	0 (0.00%)	1 (4.17%)	2 (8.33%)	1 (4.17%)	2 (8.33%)	9 (37.50%)	4 (16.67%)	4 (16.67%)	24
Usability	0 (0.00%)	1 (4.17%)	0 (0.00%)	0 (0.00%)	1 (4.17%)	2 (8.33%)	5 (20.83%)	8 (33.33%)	4 (16.67%)	3 (12.50%)	24
Range of topics	0 (0.00%)	1 (4.17%)	1 (4.17%)	1 (4.17%)	1 (4.17%)	1 (4.17%)	6 (25.00%)	6 (25.00%)	6 (25.00%)	1 (4.17%)	24

**Table 2:** Feedback Survey - The Current Status of Radiological Clinical Audit - EuroSafe Imaging Stars

Q1	Yes	No									
Were you familiar with the ESR publication - Esperanto 2019, the ESR Guide to Clinical Audit in Radiology and the ESR Clinical Audit Tool before you were sent this survey?	18 (38.30%)	29 (61.70%)									
Q2	Word of mouth /colleagues	ECR 2019	ESR email /twitter / publication	National radiology society	Other						
How did you hear about Esperanto 2019?	2 (11.11%)	13 (72.22%)	8 (44.44%)	7 (38.89%)	1 (5.56%)						
Q3	Clinical relevance	Readability	Accessibility								
Please grade the Esperanto 2019 document according to the following criteria (scale 1 = not useful, 10 = very useful)	18 (see table below*)	18 (see table below*)	18 (see table below*)								
Q4	Clinical utility	Usability	Range of topics								
Please grade the Esperanto 2019 templates according to the following criteria (scale 1 = not useful, 10 = very useful)	18 (see table below**)	18 (see table below**)	18 (see table below**)								
Q5	Yes	No	In development								
Does your radiology department engage in clinical audit - with a programme of clinical audit in place where findings, actions and re-audits are documented?	36 (76.60%)	4 (8.51%)	7 (14.89%)								
Q6	Yes	No									
Do you have a nominated department lead for clinical audit?	23 (48.94%)	24 (51.06%)									
Q7	Administrative support		Infrastructure /IT support		Clinical leadership		Clinical Engagement		Managerial support		
Which of the following local requirements for effective clinical audit are present in your department?	Yes	No	Yes	No	Yes	No	Yes	No	Yes	No	
	36 (78.26%)	10 (21.74%)	40 (86.96%)	6 (13.04%)	42 (91.30%)	4 (8.70%)	31 (68.89%)	14 (31.11%)	36 (76.60%)	11 (23.40%)	
Q8	Yes	No	In development								
Currently, does your department have a clinical audit structure in place allowing compliance with the EU-BSS?	21 (44.68%)	9 (19.15%)	17 (36.17%)								
Q9	Yes - exists, participate	No - exists, do not participate	No - does not exist								
Does your department participate in a national radiology audit programme?	21 (44.68%)	2 (4.26%)	24 (51.06%)								
Q10	National radiology society	Governmental body	Other national society	Other							
How is the national audit programme organised?	12 (25.53%)	13 (27.66%)	1 (2.13%)	21 (44.68%)							
Q11	Yes	Yes - but additional resources required	No								
In principle, would your department be prepared to participate in pan-European surveys/audits co-ordinated by the ESR/national radiology societies?	20 (42.55%)	27 (57.45%)	0 (0.00%)								
*	1	2	3	4	5	6	7	8	9	10	TOTAL
Clinical relevance	0 (0.00%)	1 (5.56%)	0 (0.00%)	0 (0.00%)	0 (0.00%)	0 (0.00%)	2 (11.11%)	10 (55.56%)	3 (16.67%)	2 (11.11%)	18
Readibility	0 (0.00%)	0 (0.00%)	0 (0.00%)	0 (0.00%)	1 (5.56%)	0 (0.00%)	1 (5.56%)	11 (61.11%)	3 (16.67%)	2 (11.11%)	18
Accessibility	0 (0.00%)	0 (0.00%)	0 (0.00%)	0 (0.00%)	0 (0.00%)	0 (0.00%)	7 (38.89%)	3 (16.67%)	5 (27.78%)	3 (16.67%)	18
**	1	2	3	4	5	6	7	8	9	10	TOTAL
Clinical utility	0 (0.00%)	0 (0.00%)	0 (0.00%)	1 (5.56%)	0 (0.00%)	0 (0.00%)	9 (50.00%)	4 (22.22%)	1 (5.56%)	3 (16.67%)	18
Usability	0 (0.00%)	0 (0.00%)	1 (5.56%)	0 (0.00%)	0 (0.00%)	1 (5.56%)	3 (16.67%)	9 (50.00%)	3 (16.67%)	1 (5.56%)	18
Range of topics	0 (0.00%)	0 (0.00%)	0 (0.00%)	1 (5.56%)	0 (0.00%)	0 (0.00%)	3 (16.67%)	10 (55.56%)	3 (16.67%)	1 (5.56%)	18

The responses were received, data cleaned, results tabulated, and feedback collated by the ESR Office, individual responses have been kept anonymous. It should be noted that both the ESR National Radiological Societies and the EuroSafe Imaging Star network contain members from within and outside of the European Union; results from both groups are included.

## Results

The results of the two surveys are recorded in Table 1 (radiology department responders via National Radiological Societies network) and Table 2 (EuroSafe Imaging Star department responders).

- A total of 43 radiology departments (28 EU, 15 outside the EU) from 16/48 National Radiological societies (12 EU, 4 outside the EU) responded to the survey distributed by the National Radiological Societies.
- A total of 47/116 EuroSafe Imaging Star departments (41% response rate, 37 EU based, 10 outside the EU) responded to the ESR-distributed second survey.
- Six out of 43 responding departments in the National Radiological Societies distributed survey declared membership of the ESR EuroSafe Imaging Star network. These departments did not participate within the ESR-distributed EuroSafe Imaging Star department survey.

The survey results are further discussed and analysed in the discussion section.

## Discussion

The development, implementation and documentation of robust processes of clinical audit are both a high clinical priority and a legal requirement for all European radiology departments. A key component in effective clinical audit is a functional infrastructure at both departmental and national level, allowing external direction (and guidance) of departmental internal audit, with the potential for wider collaborations with hub organisations such as the ESR.

A reasonable response rate (41%) was obtained for the EuroSafe Imaging Star component of the survey and allows some observations of wider European clinical audit practice and associated challenges. Only 43 departments from 16/48 National Radiological Societies responded – although these responses are valuable in themselves, the actual number of responders is small and cannot be considered representative of European practice. These latter results do lend credence however to the findings of the 2019 published National Radiological Societies survey [7] – in particular the previous survey findings show that only 22% of National Radiological Societies had an administrative facility dedicated to clinical audit, 72% had a functional means of communication with their national radiology departments and only 36% of National Radiological Societies were in regular communication with these departments.

As previously alluded to, the promotion and dissemination of good clinical audit practice is seen as a high priority by the ESR. The publication in 2019 of an updated version of Esperanto – the ESR clinical audit guide and the accompanying toolkit of 30 audit templates (with a focus on radiation protection) – was a key component of the ESR audit promotion initiative [4]. Esperanto (version 2) was launched at ECR 2019 and the clinical audit guide and toolkit were widely publicised using existing ESR networks and communications with individual, department and National Radiological Society members.

In this context, positive response rates around awareness of Esperanto and its contents of only 38% respondents (EuroSafe Imaging Stars) and 58% respondents (National Radiological Society contacted departments) are disappointing and will need further consideration and review. ESR promotional activities (email, twitter, publications and ECR-related) were the most common mechanism for alerting EuroSafe Imaging Star departments to Esperanto 2019 (21 positive responses), with 7 EuroSafe Imaging Star departments having been informed by their National Radiological Society. Departmental feedback in both surveys on both the Esperanto ESR Guide to Clinical Audit in Radiology and the ESR Clinical Audit Tool can be seen to be graded as positive/very positive by the majority of responders (see Tables 1 and 2) in terms of clinical utility, range of topics and accessibility.

The survey also evaluated whether key requirements for establishing a local clinical audit infrastructure were in place (administrative, IT, clinical leadership and engagement and managerial support). Overall departmental responses in both surveys to these questions were generally positive (the majority >70-75%).

In the EuroSafe Imaging Star department survey 36 departments (77%) and 22 departments (51%) in the National Radiological Societies departmental survey) have a clinical audit programme in place. However, interestingly, this positive response rate drops to only 21 departments (44.7%) in the EuroSafe Imaging Star department survey and 11 departments (25.6%) in the National Radiological Societies departmental survey when asked if the existing departmental clinical audit structure will allow BSSD compliance (noting work in progress in this area in 17/47 EuroSafe Imaging Star departments). The recorded positive response rate of 44.7% is significantly lower than that obtained for a similar question in the previous ESR survey examining EuroSafe Imaging Star department BSSD compliance [6], where 82% of departments responded positively (54/66). Acknowledging a smaller return rate for the current survey, it is feasible that in the previous survey some departments may have overestimated the capacity of their audit systems to allow BSSD compliance. As the challenges and complexities of BSSD implementation have subsequently become more apparent this may have served to highlight existing limitations in supporting audit infrastructure.

The final survey questions related to national clinical audit programmes. In the EuroSafe Imaging Star survey 21/47 departments have access to a national radiology audit programme organised via the relevant National Radiological Society (two departments do have access but do not participate). Levels of participation in a national audit programme were higher in the National Radiological Societies departmental survey (27/43, 62.8% departmental participation) although, as discussed, these results may not be representative of wider practice.

All departments in the EuroSafe Imaging Stars survey (and the majority in the National Radiological Societies departmental survey) expressed willingness to participate in larger-scale, pan-European surveys potentially co-ordinated by ESR/National Societies. It is important to note that the majority of EuroSafe Imaging Star responders (57.4%) felt that additional resources would be required to facilitate this participation.

## Conclusion

Although the EuroSafe Imaging Star survey had a reasonable response rate, the survey distributed by the National Radiological Societies had returns from only 43 radiology departments from a minority of National Radiological Societies. There are likely to be many factors involved in this low rate of return, but underdeveloped communication mechanisms and infrastructure between National Radiological Societies and their radiology departments are likely to be important factors. This may also be reflected in the relatively low prevalence of national audit programmes available for departmental participation.

Both surveys revealed relatively low awareness of Esperanto, the ESR Guide to Clinical Audit in Radiology and the ESR Clinical Audit Tool and also a lack of existing clinical audit processes to allow BSSD compliance, despite apparently well-developed local clinical audit infrastructures.

Continuing investment and resource allocation in the development of local and national clinical audit infrastructure is required, allied with increased understanding and acceptance of the importance of clinical audit at departmental and governmental level. Key stakeholders such as the ESR, National Radiological and specialist Societies will be fundamental in the required continuing promotion and integration of clinical audit into European radiological practice.

## Data Availability

All data generated or analysed during this study are included in this published article.
